# Clinical practice improvement in the genetics clinic

**DOI:** 10.1186/1897-4287-10-S2-A44

**Published:** 2012-04-12

**Authors:** S Greening

**Affiliations:** 1 Wollongong Hereditary Cancer Clinic, Australia

## 

Quality in health is defined as ‘doing the right thing, the first time, in the right way, and at the right time”. A model for practical quality improvement in the genetic unit will be presented, along with tools and techniques relevant to the practice of clinical genetics, and examples from real life practice.

Clinical Improvement follows five phases, including:

• Project Phase; the process that needs improving is highlighted and defined, a team is formed, an aim or mission statement is produced, and a method of measurement is chosen.

• Diagnostic Phase: collect the evidence needed to diagnose the problem (its nature, its causes), and organise and prioritise the information using specific templates.

• Intervention Phase: involves using PDSA cycles to trial efficient methodologies and refine an implementation plan.

• Impact Phase: implement the changes, including implementation of a resistance management plan

• Sustaining Improvement phase; develop plans for standardisation, documentation, measurement, review and training/education of staff.

In addition, preparation of results for submission to area health Quality Awards is reviewed, and preparation of results for publication.

